# Glucocorticoid use in paediatric posterior fossa tumour surgery and the occurrence of postoperative speech impairment

**DOI:** 10.1007/s00381-025-06850-0

**Published:** 2025-07-11

**Authors:** Rebekka Sarup, Aske F. Laustsen, Martin K. Sørensen, Conor Mallucci, Barry Pizer, Kristian Aquilina, Emanuela Molinari, Magnus Aasved Hjort, Radek Frič, Per Nyman, Magnus Sabel, Pelle Nilsson, Algimantas Matukevičius, Peter Hauser, Katalin Mudra, Andrea Carai, Julian Zipfel, Eelco Hoving, Kirsten van Baarsen, Vladimír Beneš IIIrd, Andreas Peyrl, Karsten Nysom, Astrid Marie Sehested, Kjeld Schmiegelow, Marianne Juhler, Jonathan K. Grønbæk, René Mathiesen

**Affiliations:** 1 Department of Neurosurgery, The University Hospital Rigshospitalet, Blegdamsvej 9, DK-2100 Copenhagen E, Denmark; 2 Department of Paediatrics and Adolescent Medicine, The University Hospital Rigshospitalet, Blegdamsvej 9, DK-2100 Copenhagen E, Denmark; 3 Department of Neuroanesthesiology, The University Hospital Rigshospitalet, Blegdamsvej 9, DK-2100 Copenhagen E, Denmark; 4 Institute of Clinical Medicine, Faculty of Medicine, University of Copenhagen, Blegdamsvej 3B, DK-2200 Copenhagen, Denmark; 5 Department of Paediatric Neurosurgery, Alder Hey Children’s NHS Foundation Trust, E Prescot Rd, Liverpool, L14 5 AB UK; 6 Department of Paediatric Oncology, Alder Hey Children’s NHS Foundation Trust, Liverpool, UK; 7 Department of Neurosurgery, Great Ormond Street Hospital for Children, Great Ormond Street, London, WC1 N 3 JH UK; 8 Department of Neurology, The Queen Elizabeth University Hospital, University of Glasgow, University Avenue, Glasgow, G12 8QQ UK; 9 Department of Pediatric Hematology and Oncology, St Olavs Hospital, Postboks 3250 Torgarden, 7006 Trondheim, Norway; 10 Department of Neurosurgery, Oslo University Hospital, Postboks 4950 Nydalen, Oslo, 0424 Norway; 11 Department of Biomedical and Clinical Sciences, Crown Princess Victoria Children’s Hospital, Linköping University, Linköping, Sweden; 12 Department of Pediatrics, Institute of Clinical Sciences, Sahlgrenska Academy, University of Gothenburg, Gothenburg, Sweden; 13 Childhood Cancer Centre, Silvia Children’s Hospital, Sahlgrenska University Hospital, Gothenburg, Queen Sweden; 14 Department of Neuroscience, Section of Neurosurgery, Uppsala University, Uppsala, Sweden; 15 Department of Neurosurgery, Lithuanian University of Health Sciences (LUHS), Kaunas, Lithuania; 16 2nd Department of Pediatrics, Semmelweis University, Tűzoltó U. 7-9, 1094 Budapest, Hungary; 17 Velkey László Child’s Health Center, BAZ County Central Hospital and University Teaching Hospital, Szentpéteri Kapu 72-76, 3526 Miskolc, Hungary; 18 Department of Neurosciences, Neurosurgery Unit, Bambino Gesù Children’s Hospital, IRCCS, Rome, Italy; 19 Department of Neurosurgery, Pediatric Neurosurgery, University Hospital Tuebingen, Hoppe-Seyler-Str. 3, 72076 Tuebingen, Germany; 20 Princess Máxima Center for Pediatric Oncology, Heidelberglaan 25, 3584 CS Utrecht, The Netherlands; 21 Department of Neurosurgery, Second Faculty of Medicine, Charles University and Motol University Hospital, Prague, Czech Republic; 22 Department of Pediatrics and Adolescent Medicine, Medical University of Vienna, Waehringer Guertel 18-20, 1090 Vienna, Austria; 23 Department of Neurosurgery, Aarhus University Hospital, Palle Juul-Jensens, Boulevard 99, 8200 Aarhus, Denmark

**Keywords:** Cerebellar mutism syndrome, Neurosurgery, Brain neoplasm, Preoperative, Intraoperative, Postoperative speech impairment

## Abstract

**Purpose:**

Postoperative speech impairment (POSI) is a core symptom of cerebellar mutism syndrome (CMS) and is a common complication after the resection of paediatric posterior fossa (PF) tumours. Preoperative glucocorticoids (pGC) are considered standard treatment to reduce tumour oedema; in addition, glucocorticoids are often administered intraoperatively (iGC) to reduce both postoperative nausea and vomiting. The study aims to investigate whether the occurrence of POSI may be associated with pGC and iGC.

**Methods:**

In a prospective observational multicentre study, we included children with a PF tumour requiring either resection or open biopsy. The use of pGC and iGC, including drug type and dose, was registered. Postoperative speech status was classified as mutism, reduced speech, or habitual speech, where mutism and reduced speech were considered POSI of higher and lower severity, respectively. Proportional odds logistic regression with adjustment for tumour type, tumour location, and age was used to analyse the occurrence of POSI associated with glucocorticoids (GC).

**Results:**

From August 2014 to November 2024, we recruited 810 children, of whom 605 were included in the primary analysis. We found no association between the use of GC (pGC nor iGC) and the occurrence of POSI. The result did not change when adjusting for tumour type, tumour location, and age. The analysis included both a comparison between using and not using pGC (OR 1.06 [95% CI 0.46 –2.49], reference level: use of pGC) and/or iGC (1.28 [0.58–2.82], reference level: use of iGC), and a dose–response analysis of the occurrence of POSI in relation to doubling the dose of GC (pGC OR 1.28 [0.84–1.98]; iGC OR 1.07 [0.62–1.82]).

**Conclusion:**

Our study did not find evidence of a significant change in the occurrence of POSI with the use of pGC or iGC, but our results alone cannot rule out that the administration of pGC or iGC may have some effect. Therefore, our data do not call for a change in recommendations for the use of GC as protection against the development of POSI.

Trial registration number: Clinicaltrials.gov (NCT02300766).

Date of registration: November 25, 2014

**Supplementary information:**

The online version contains supplementary material available at 10.1007/s00381-025-06850-0.

## Introduction

Cerebellar mutism syndrome (CMS) occurs after tumour resection in the posterior fossa (PF) with a reported incidence ranging from 8 to 39% [1]. The cardinal symptom of CMS is postoperative speech impairment (POSI), defined as reduced speech or mutism. Additional symptoms include emotional lability, hypotonia, dysphagia, and ataxia [2]. 

Strong risk factors for POSI include medulloblastoma, midline tumour location, and lower age [3]. Known risk factors of CMS are all predetermined at the time of surgery, highlighting the need to identify modifiable risk factors.

Proximal disruption of the dentato-thalamo-cortical pathway (DTCp) is strongly implied to play a central role in the development of CMS, although the full aetiology of the syndrome is unknown [4–7]. The DTCp originates from the dentate nucleus and ascends through the superior cerebellar peduncle (SCP). The fibres decussate in the midbrain tegmentum to synapse in the contralateral thalamus and terminate in the primary motor cortex and associated pre-motor areas, including the supplementary motor area, premotor cortex, and prefrontal cortex [8]. Glucocorticoids (GC) are routinely given preoperatively to reduce peritumoural oedema and intraoperatively to reduce postoperative nausea and vomiting [9]. Side effects, such as delayed wound healing, glucose intolerance, immunosuppression, fluid retention, and electrolyte disturbances, are the main challenges associated with GC use [10]. However, their efficacy at reducing peritumoural oedema has the potential to both relieve symptoms of raised intracranial pressure and prevent mechanical harm to critical anatomical structures of the cerebello-cerebral circuit, like the SCP, and hypothetically may lead to a decreased occurrence of POSI.

## Methods

### Study population

The European Study of the Cerebellar Mutism Syndrome is an observational, prospective multicentre study and is registered on Clinicaltrials.gov (NCT02300766). The full protocol approved by the Research Ethics Committee of the Capital Region of Denmark has previously been published [1]. We included paediatric patients (< 18 years old) with a tumour in the PF undergoing tumour resection or open biopsy. The patients underwent the neurosurgical procedure at one of the 35 collaborating centres in 15 different countries.

### Data collection and management

Data were collected by either a paediatrician or a neurosurgeon at distinct time points: preoperatively, < 72 h, and 2 weeks postoperatively. In case of emergency surgery, the protocol allowed patient inclusion postoperatively within 7 days.

Basic information regarding sex, height, and weight were collected preoperatively. Age was defined as age at surgery. In patients where the date of surgery was not available, age was estimated at the planned surgery date or the date of diagnosis instead.

The primary outcome was POSI assessed 2 weeks postoperatively. POSI was considered at two severity levels, as either mutism or reduced speech. *Mutism* was defined as a complete lack of speech with an inability to produce words or short sentences, while sounds such as whining or crying could still be present. *Reduced speech* was defined as speech restricted to isolated words or short sentences despite vigorous stimulation.

GC data were gathered at the 2-week follow-up. Administration of preoperative glucocorticoids (pGC) and intraoperative glucocorticoids (iGC) was recorded. The preoperative period was defined as the period from diagnosis until the start of surgery, and the intraoperative period from the start of surgery until wound closure. Dexamethasone, betamethasone, prednisone/prednisolone, and methylprednisolone were the standard options in the database, but the clinician could document other types of GC, if needed (Supplementary materials). For each of the GC types, we gathered the maximum dose (mg/day) preoperatively, while iGC exposure was registered as the total dose (mg). The original doses were converted into prednisolone equivalent doses by the conversion factor of 0.75 for betamethasone and dexamethasone, 4 for methylprednisolone, and 20 for hydrocortisone for every 5 prednisolone [11, 12]. Doses for each patient were determined by clinicians according to local standards. Postoperative GC use was also registered but not included in our analysis due to the early onset of POSI within 0–3 days, rendering uncertainty about the existence of a time interval between postoperative GC and the onset of POSI [13].

Tumour histology was determined by the local department of pathology and registered by the investigator as either pilocytic/pilomyxoid astrocytoma, medulloblastoma, ependymoma, atypical teratoid rhabdoid tumour (AT/RT), or other.

Tumour location was registered by the neurosurgeon within 72 h postoperatively, with the option to record multiple locations. Based on this registration, we assigned the location as either one of four separate categories: (1) brainstem; (2) fourth ventricle, with no brainstem involvement; (3) cerebellar vermis, with neither brainstem nor fourth ventricle location; or (4) cerebellar hemisphere, with neither brainstem, fourth ventricle, nor cerebellar vermis location.

### Statistical analysis

We analysed the occurrence of POSI using proportional odds logistic regression. The outcome variable was speech status recorded within the first 2 weeks postoperatively, with three ordered levels in ascending order: habitual speech (0), reduced speech (1), or mutism (2).

We did *uniaspect analyses* of pGC and iGC separately. Uniaspect refers to the comparison between receiving GC and not receiving GC and including dose to explore a possible dose–response relationship. Doses were adjusted for weight (mg/kg). Since GC doses had a right-skewed distribution instead of a normal distribution, a logarithmic transformation compared to no transformation of doses was explored. The logarithmic transformation resulted in a better fit of the data and was therefore chosen. The odds ratios (OR) for dose depict the change in odds of POSI for every doubling in dose. The subsequent model included pGC and iGC *mutually adjusted*. We hypothesised that pGC may confound the iGC effect on POSI, as the use or dose of pGC may influence the decision to continue GC or the dose given intraoperatively (Table 1, Supplementary materials).

GC use and dose could be confounded by the physician’s knowledge of POSI risk factors, namely tumour location and characteristics on preoperative neuroimaging. Therefore, we did a stepwise inclusion of tumour type and then location (Table 2, Supplementary materials), but both were included in the final regression model (Table 2).

The final model was *adjusted for age* as a possible confounder since an increase in age is associated with a decrease in the risk of POSI [3].

Earlier results from the European Cerebellar Mutism Syndrome study indicated a higher prevalence of unknown postoperative speech status in children aged 0–2 years compared to 3–6 and 7–17 years. To consider potential uncertainty in determining speech in young children, the effects of excluding patients younger than 2 years were explored and did not alter our results (Table 3, Supplementary data). We investigated the change in the proportional odds estimates explained by missing data induced by adding new variables in each step of the analysis. Missing data did not have any major effect on model estimates but did contribute to an increase in the odds of POSI with not receiving pGC in the model adjustment for tumour type and location (Table 4, Supplementary materials).

To explore the proportional odds assumption, the ordinal logistic regression estimates were compared with the logistic regression of speech status levels 0 vs. 1 or 2, and 0 or 1 vs. 2 (Table 5, Supplementary data). The proportional odds assumption was tested using a Brant test, and the assumption held for our model.

GC outliers were defined as log-transformed doses more than or less than 3 standard deviations from the mean of the log-transformed. Although the removal of the outlier doses did not change the overall result of the analysis, they were removed from the final analysis to exclude extreme doses with little clinical relevance (Table 6, Supplementary materials).

All results were reported with a 95% confidence interval and were considered significant for *p*-value < 0.05.

The statistical analyses were performed in the statistical software R version 4.4.1.

## Results

**Fig. 1:**
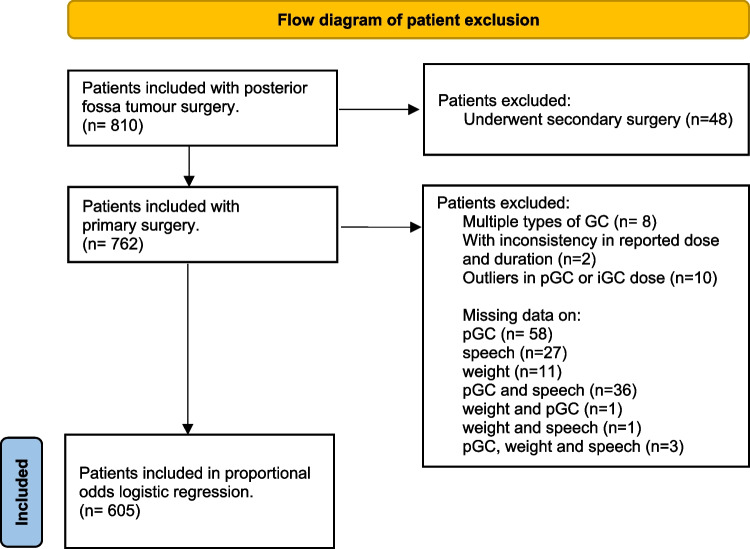
Flow diagram of patient exclusion

The study recruited 810 patients from August 2014 to November 2024. Forty-eight patients underwent secondary surgery at the time of inclusion. As a result, 762 patients with primary surgery were included for further characterisation (Fig. 1).

In the full cohort (*n* = 762), GC was given to 546 patients (72%) preoperatively and 327 patients (43%) intraoperatively. The use of GC was unknown for 61 patients (8%) preoperatively and 111 patients (15%) intraoperatively.

Dose was unknown for 100 patients (13%) preoperatively and 144 patients (19%) intraoperatively. The median dose was 1.7 mg/kg/day (min 0.1, max 55.7) for the preoperative period and 1.1 mg/kg (min 0.01, max 22.2) for the intraoperative period (Table 1). We investigated combinations of the use of pGC and iGC in patients with known doses. We observed that using pGC without iGC (*n* = 228) and using both pGC and IGC (*n* = 226) were the most common combinations. There was no use of either pGC or iGC in 91 patients, and iGC only was used in 55 (Table 2).

**Table 1:** Patient characteristics

	Full cohort ***N*** = 762	GC cohort ***n*** = 680	Mutism ***n*** = 82	Reduced speech ***n*** = 92	Habitual speech ***n*** = 475	Unknown speech ***n*** = 31
**Sex** *n *(%)						
Male	432 (57%)	386 (89%)	46 (12%)	53 (14%)	270 (70%)	17 (4%)
Female	330 (43%)	294 (89%)	36 (12%)	39 (13%)	205 (70%)	14 (5%)
**Age in years** Median (IQR) [min–max]	7.0 (7.1) [0–17.9]	7.0 (7.0) [0–17.9]	4.5 (6.2) [0.9–16.1]	6.3 (4.9) [0.5–17.6]	7.7 (7) [0–17.9]	6.0 (8.9) [0.1–16.1]
**Tumour type** *n *(%)						
Pilocytic/ pilomyxoid astrocytoma	297 (39%)	281 (95%)	15 (5%)	27 (10%)	232 (83%)	7 (2%)
Medulloblastoma	217 (28%)	206 (95%)	41 (20%)	39 (19%)	115 (56%)	11 (5%)
Ependymoma	65 (9%)	60 (92%)	12 (20%)	9 (15%)	35 (58%)	4 (7%)
AT/RT	20 (3%)	20 (100%)	5 (25%)	4 (20%)	8 (40%)	3 (15%)
Other	67 (9%)	65 (97%)	5 (8%)	9 (14%)	51 (78%)	0 (0%)
Unknown	96 (13%)	48 (50%)	4 (8%)	4 (8%)	34 (71%)	6 (12%)
**Tumour location** *n *(%)						
Fourth ventricle	143 (19%)	129 (90%)	25 (19%)	24 (19%)	74 (57%)	6 (5%)
Brainstem	258 (34%)	232 (90%)	49 (21%)	41 (18%)	129 (56%)	13 (6%)
Cerebellar vermis	135 (18%)	125 (93%)	2 (2%)	15 (12%)	103 (82%)	5 (4%)
Cerebellar hemisphere	175 (23%)	152 (87%)	2 (1%)	9 (6%)	137 (90%)	4 (3%)
Unknown	51 (7%)	42 (82%)	4 (10%)	3 (7%)	32 (76%)	3 (7%)
**pGC** *n *(%)						
No	155 (20%)	155 (100%)	12 (8%)	15 (10%)	123 (79%)	5 (3%)
Yes, dose known	507 (67%)	507 (100%)	69 (14%)	75 (15%)	338 (67%)	25 (5%)
Yes, dose unknown	39 (5%)	13 (33%)	0 (0%)	1 (8%)	12 (92%)	0 (0%)
Unknown	61 (8%)	5 (8%)	1 (20%)	1 (20%)	2 (40%)	1 (20%)
Prednisolone Eq. max dose (mg/kg/pr. day) Median (Q1–Q3) [min–max]	1.7 (1.1–2.5) [0.1–55.7]	1.7 (1.1–2.5) [0.1–55.7]	1.8 (1.4–2.7) [0.1–8.0]	2 (1.4–2.7) [0.5–6.7]	1.5 (1.1–2.4) [0.1–55.7]	1.7 (1.0–2.4) [0.1–4.0]
**iGC** *n *(%)						
No	324 (43%)	324 (100%)	37 (11%)	50 (15%)	226 (70%)	11 (3%)
Yes, dose known	294 (39%)	294 (100%)	36 (12%)	39 (13%)	205 (70%)	14 (5%)
Yes, dose unknown	33 (4%)	9 (27%)	1 (11%)	0 (0%)	8 (89%)	0 (0%)
Unknown	111 (15%)	53 (48%)	8 (15%)	3 (6%)	36 (68%)	6 (11%)
Prednisolone Eq. max dose (mg/kg) Median (Q1–Q3) [min–max]	1.1 (0.8–1.7) [0.01–22.2]	1.1 (0.8–1.7) [0.01–22.2]	1.3 (1.0–2.1) [0.5–22.2]	1.2 (0.7–1.7) [0.3—10.7]	1 (0.8–1.6) [0—19.2]	1 (0.7–1.3) [0.1—2.8]

Data for categorical variables are in *n* (% of full cohort) in the 1^st^ column, *n* (% of the specific subgroup of full cohort) in the 2^nd^ column, and *n* (% of the specific subgroup of the GC cohort) in the 3^rd^–6^th^ columns. A reduced cohort “GC cohort” was defined as patients with any known data on either pGC or iGC dose to investigate selection bias. Data for continuous variables are in median (Q1–Q3) [min–max]

**Table 2:** Use of intra- and preoperative glucocorticoids in relation to patient characteristics

	No use of pGC and iGC ***n*** = 91	No use of pGC, use of iGC with dose known ***n*** = 55	No use of iGC, use of pGC with dose known ***n*** = 228	Use of both pGC and IGC with dose known ***n*** = 226
**Sex** *n *(%)				
Male	52 (57%)	29 (53%)	131 (57%)	133 (59%)
Female	39 (43%)	26 (47%)	97 (43%)	93 (41%)
**Age in years** Median (Q1–Q3) [min–max]	7.2 (4.1–10.9) [0.003–17.9]	9.8 (6.5–13.2) [1.4–17.7]	6.8 (3.6–10.2) [0.003–17.4]	6.5 (3.1–10.1) [0.5–17.8]
**Tumour type** *n *(%)				
Pilocytic/pilomyxoid astrocytoma	40 (44%)	26 (47%)	99 (43%)	80 (35%)
Medulloblastoma	22 (24%)	9 (17%)	70 (31%)	81 (36%)
Ependymoma	6 (7%)	5 (9%)	19 (8%)	27 (12%)
AT/RT	5 (5%)	0 (0%)	7 (3%)	6 (3%)
Other	9 (10%)	11 (20%)	17 (8%)	18 (8%)
Unknown	9 (10%)	4 (7%)	16 (7%)	14 (6%)
**Tumour location,** *n *(%)				
Fourth ventricle	27 (29%)	11 (20%)	83 (36%)	86 (38%)
Brainstem	12 (13%)	13 (24%)	44 (19%)	45 (20%)
Cerebellar vermis	18 (20%)	10 (18%)	40 (18%)	45 (20%)
Cerebellar hemisphere	26 (29%)	14 (25%)	50 (22%)	43 (19%)
Unknown	8 (9%)	7 (13%)	11 (5%)	7 (3%)
**Postoperative speech status**				
Habitual speech	69 (76%)	46 (84%)	154 (68%)	148 (65%)
Reduced speech	11 (12%)	4 (7%)	38 (17%)	34 (15%)
Mutism	7 (8%)	4 (7%)	29 (13%)	32 (14%)
Unknown	4 (4%)	1 (2%)	7 (3%)	12 (5%)

Data for categorical variables are in *n* (% column). Data for continuous variables are in median (Q1–Q3) [min–max] for the subgroup specified in the column

**Fig. 2:**
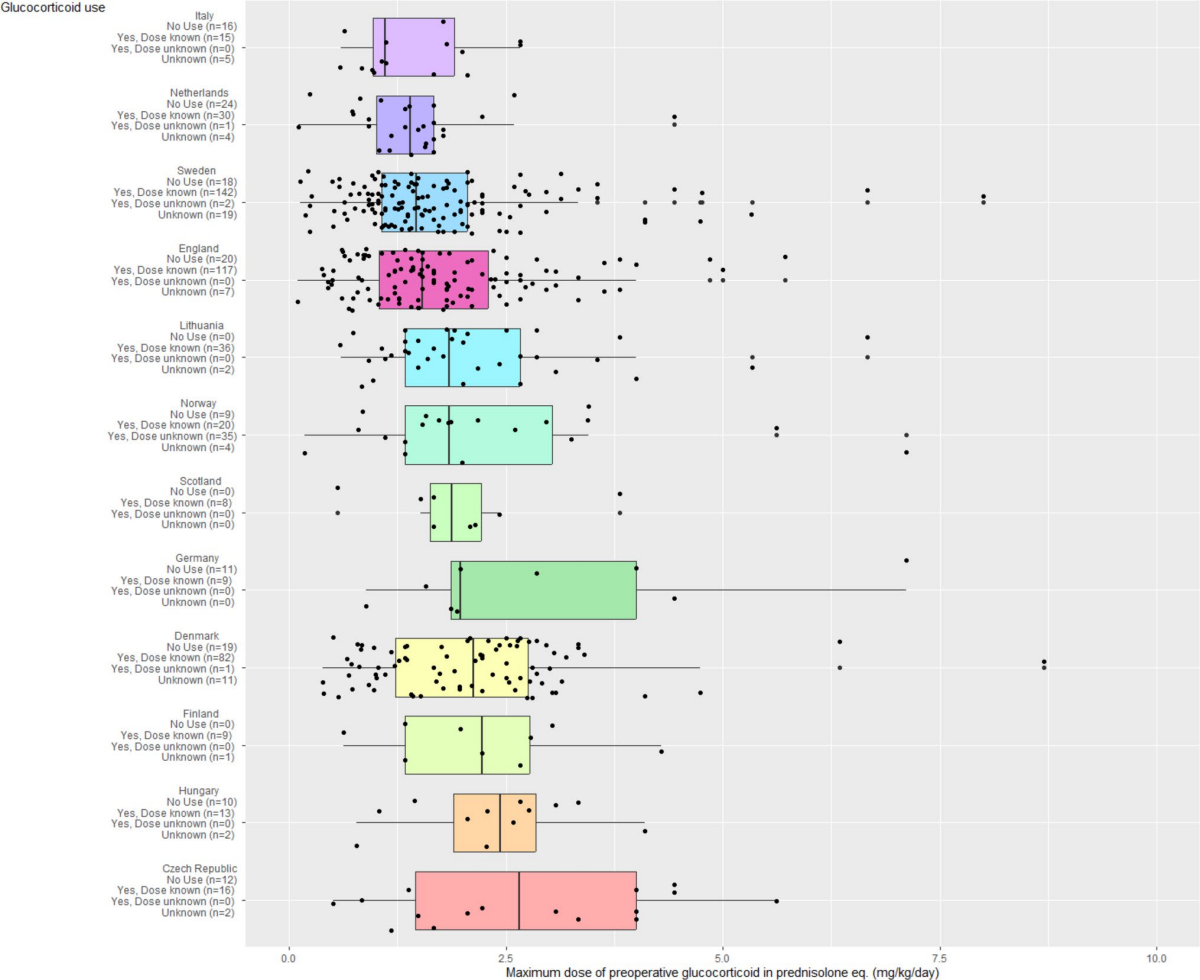
Preoperative glucocorticoid use grouped by country

We stratified GC use and dose based on countries. The lowest non-zero median pGC dose of 1.1 mg/kg/day was seen in Italy (*n* = 12) and the highest dose of 2.6 mg/kg/day was seen in the Czech Republic (*n* = 16). However, the GC use in the three countries (Sweden, England, Denmark) contributing 338 (67%) patients who received pGC was similar (1.5, 1.5, 2.1 mg/kg/day respectively) (Fig. 2).

**Fig. 3:**
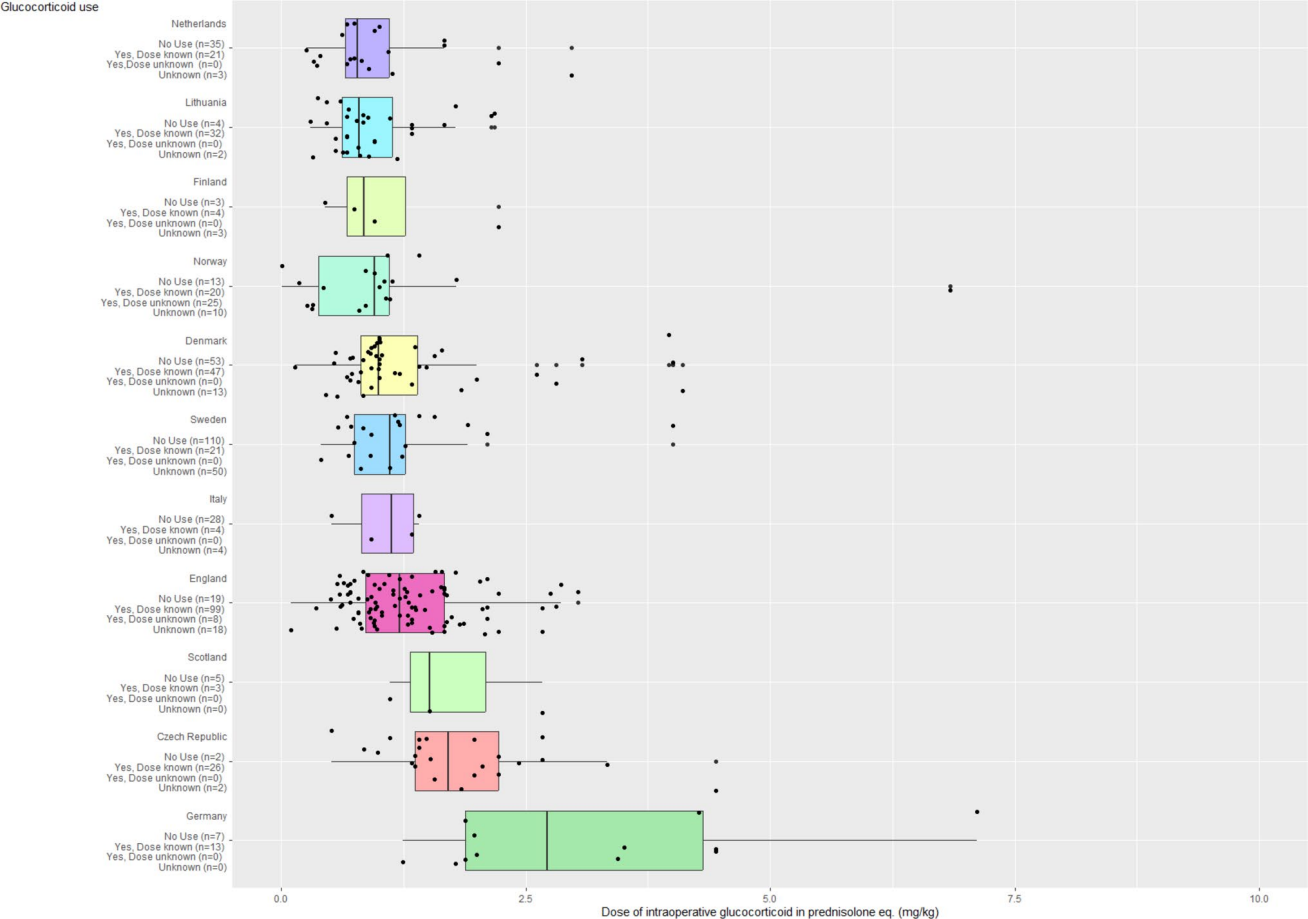
Intraoperative glucocorticoid use grouped by country

The majority of patients were included in Denmark, Sweden and England, where use of pGC was similar. Patients from Germany had a higher iGC dose than all other countries. The median iGC dose in Germany was approximately three times larger than the median dose of the lowest median dose in Lithuania. The majority of German patients did not receive pGC, while the majority of German patients did receive iGC (Fig. 3).

In the uniaspect analysis of pGC, a doubling in dose was associated with an increase of POSI (OR 1.69, [95% CI 1.17–2.46]), but we could not show a significant effect of not receiving pGC (1.16 [0.56–2.43]). Receiving no iGC (OR 1.61, [95% CI 0.79–3.32]) or doubling in iGC dose (OR 1.49, [95% CI 0.89–2.48]) was not associated with an increase in the occurrence of POSI. After adjusting for tumour type, tumour location, and age, no significant change in the occurrence of POSI was shown with no pGC use (1.06 [0.46–2.49]), pGC dose (1.28 [0.84–1.98]), no iGC use (1.28 [0.58–2.82]), or iGC dose (1.07 [0.62–1.82]) (Tables 3 and 4).

**Table 3:** Proportional odds logistic regression analysis of POSI

	Uniaspect analysis		Model 3: Adjusted analysis^a^ *n* = 485	
	OR (95% CI)	*p*-value	OR (95% CI)	*p*-value
**pGC**	*n* = 605			
No pGC given	1.16 (0.56–2.43)	0.69	1.06 (0.46–2.49)	0.89
**iGC**	*n* = 564			
No iGC given	1.61 (0.79–3.32)	0.19	1.28 (0.58–2.82)	0.53

Data are in OR (95% CI) and *p*-values. All dose values were log_2_ transformed. The uniaspect analysis was adjusted for dose of GC

^a^The analysis was adjusted for pGC, iGC, tumour type, tumour location, and age

**Table 4:** Proportional odds logistic regression analysis of POSI

	Uniaspect analysis		Model 3: Adjusted analysis^a^ *n* = 485	
	OR (95% CI)	*p*-value	OR (95% CI)	*p*-value
**pGC**	*n* = 605			
Pr. doubling in dose prednisolone equivalent maximum dose pr. day (mg/kg/day)	1.69 (1.17–2.46)	0.01	1.28 (0.84–1.98)	0.25
**iGC**	*n* = 564			
Pr. doubling in prednisolone equivalent dose (mg/kg)	1.49 (0.89–2.48)	0.13	1.07 (0.62–1.82)	0.79

Data are in OR (95% CI) and *p*-values. All dose values were log_2_ transformed

^a^The analysis was adjusted for pGC, iGC, tumour type, tumour location, and age

## Discussion

In this study, we investigated the occurrence of POSI associated with pGC and iGC in a large prospective multicentre cohort. Our main finding was that the occurrence of POSI was not significantly associated with pGC or iGC. Still, the CI’s upper bound for not giving pGC suggests up to a 2.5-fold increase in the odds of POSI compared with a 0.5-fold reduction for the lower bound. This may suggest a potentially larger protective effect than harmful effect of pGC, but a significant association could not be determined. Similarly, the estimates of no iGC use had a CI upper bound of 2.8 and a lower bound of 0.6.

It was unexpected that no significant association between the occurrence of POSI and pGC or iGC was found, since our hypothesis was based on the key pathophysiological mechanism implied in CMS, that is, that POSI occurrence could be reduced by preventing mechanical harm of peritumoural oedema to the efferent pathway of DTCp. Our findings seem to suggest that the unmodifiable risk factors of tumour type, tumour location, and age outweigh the impact of pGC or iGC on the occurrence of POSI. This agrees with the current literature view on POSI as a postoperative complication mainly predicted by risk factors that are predetermined at the time of surgery. Although pGC and iGC may not be able to fully prevent POSI, it remains unanswered whether pGC and iGC are associated with the duration of POSI. At the same time, the nuances of speech impairment are not fully captured by a categorical scale with three severity levels, and future analyses of speech samples from our cohort may provide new insights. We investigated the association of the occurrence of POSI with pGC and iGC use, but the use of GC may also alter the occurrence or severity of other symptoms of CMS such as emotional lability, ataxia, and hypotonia. Future studies should evaluate these associations as well, since a single symptom such as POSI is not fully representative of CMS.

To our knowledge, our study is the first to investigate the relationship between POSI and pGC or iGC exposure. In a previous study, iGC did not affect the occurrence of postoperative complications in paediatric elective neurosurgery [14]. Postoperative complications were assessed as a combined group of events, including death, respiratory complications, infections, coma, seizure, and any new postoperative neurological deficits. It would be valuable to know how GC was associated with the reduction of existing and new neurological deficits after surgery in another paediatric cohort, but as neither POSI nor CMS was specified in this study, it is not possible to compare these results with the observations from our study.

Our study provides a unique insight into the differences and similarities in GC use and dosing in paediatric PF tumour surgery across Europe. The variation in GC use and dose may reflect the lack of unanimous treatment guidelines, as reported in previous literature [15, 16]. pGC is primarily given to reduce symptoms of high intracranial pressure caused by peritumoural oedema. On the other hand, iGC use can reflect the continuation of pGC, but is also given solely to reduce surgical oedema and/or postoperative nausea and vomiting. In our study, we observed the GC treatment as selected and administered by clinicians in different centres, but we did not document the specific clinical indication for GC treatment for the individual patient.

The sample sizes in some participating centres were relatively small, and this should be taken into consideration, as the GC use in these centres may not be representative of the respective countries. Doses may reflect regional differences in GC administration policies, although missing data and the heterogeneity of patients in our study cohort may further contribute to the variance in GC regimens. Our study had few eligibility criteria; and thus, our cohort was characterized by a broad age range, diverse tumour subtypes, and the fact that both emergency and elective surgeries were included.

The paediatric neurosurgical and neuro-oncological community continues to call for evidence-based guidelines for GC regimens. This necessitates a discussion on what types of studies should be prioritized and the feasibility of these studies. Surveys, working groups, and earlier retrospective studies have highlighted some of the factors that may influence how clinicians use GC and explanations for variability in practice [17–21]. This includes factors such as whether patients had elective or acute surgeries, supratentorial or infratentorial tumour location, severity of symptoms of raised intracranial pressure, and presence of neurological deficits, amount of peritumoral oedema, and presence of obstructive hydrocephalus. Randomization of critically ill paediatric patients poses ethical considerations, as GC use is widely accepted as a treatment that is safe in short durations and an effective treatment for alleviating symptoms of raised intracranial pressure. While randomization between placebo or GC treatment may be too controversial, trials investigating optimal dosing regimens remain necessary. The factors highlighted by previous studies could be barriers for clinicians to include patients in a clinical trial and must be weighed carefully, such that future study design does not compromise patient safety and that the randomization of GC dose would not delay other necessary interventions. Furthermore, with such rare and heterogeneous diseases such as paediatric CNS tumours, it is necessary to establish protocols for clinical trials of GC treatment through international collaboration and prioritize outcome measures that could challenge and improve current practice.

## Limitations and strengths

Our analysis did account for known risk factors of POSI, but we cannot exclude the possibility of other important factors that may have impacted our results. Due to the observational study design, and thus lack of treatment randomization, there is a risk of confounding by indication. Any changes in the occurrence of POSI associated with GC may reflect the underlying indication for GC treatment, e.g. severity of peritumoural oedema, which may differ between different tumour types, rather than the effect of GC on POSI. Unfortunately, our study did not yield sufficient statistical power for subgroup analyses stratifying by tumour type. We did, however, observe a tendency towards a more abundant use of especially pGC in patients with medulloblastoma compared with patients with pilocytic astrocytoma (Table 2).

We considered the opportunity that treatment effects may differ between patient subgroups. Preoperative hydrocephalus is common among patients with PF tumours. Hydrocephalus can be treated with a shunt, EVD, ventriculostomy, or no preoperative treatment as tumour resection can also be considered primary decompressive treatment. It may be that the effect of pGC differs depending on whether patients received a shunt together with pGC. Unfortunately, we did not have the power to do any subgroup analysis. A potential confounder that was not accounted for is the use of other anti-oedema agents, such as mannitol and hypertonic saline: due to the study design, however, it is unknown whether patients received these agents pre- or intraoperatively. If patients with no pGC received another anti-oedema treatment, the beneficial effects of pGC may have been concealed. Postoperative GC was registered but not included in our analysis, since the relationship between postoperative GC and the occurrence of POSI is problematic to investigate due to the early postoperative onset of POSI (within 0–3 days) [13]. Considering this and the long biological half-lives of GC makes a direct causal mechanism between postoperative GC and the occurrence of POSI implausible. Still, postoperative GC could potentially modulate the severity or duration of POSI or other CMS symptoms. The strengths of our study are its prospective design and the large population size across multiple institutions, which largely limits selection bias and allowed us to adjust for known risk factors as confounders when studying the association between POSI and GC.

## Conclusion

Our study did not find evidence of a significant change in the occurrence of POSI with the use of pGC or iGC, but our results alone cannot rule out that the administration of pGC or iGC may have some effect. Therefore, our data do not call for a change in recommendations for the use of GC to protect against the development of POSI. We document both the heterogeneity and similarities of GC treatment in paediatric PF tumour surgery across Europe, emphasizing the difficulties of investigating the effect of GC on an early postoperative outcome. Future studies can examine if GC changes the duration of POSI.

## Supplementary information

Below is the link to the electronic supplementary material.

ESM 1 (DOCX 70.1 KB)

## Data Availability

No datasets were generated or analysed during the current study.

## Abbreviation

CI: Confidence interval
DTCp: Dentato-thalamo-cortical pathway
GC: Glucocorticoids
iGC: Intraoperative glucocorticoids
OR: Odds ratio
PF: Posterior fossa
pGC: Preoperative glucocorticoids
POSI: Postoperative speech impairment
SCP: Superior cerebellar peduncle
