# Supplementary material for: Glucocorticoid use in paediatric posterior fossa tumour surgery and the occurrence of postoperative speech impairment
Source: Childs Nerv Syst. 2025 Jul 11;41(1):231. doi: 10.1007/s00381-025-06850-0 (PMC12254087; doi:10.1007/s00381-025-06850-0)
Supplement: Supplementary file 1 — (DOCX 70.1 KB) [file 381_2025_6850_MOESM1_ESM.docx]

**Supplementary materials**

**Table a: Overview of different types of glucocorticoids used in this study**

**Table b: Proportional odds logistic regression with estimates of confounding variables**

**Table c: Step-wise inclusion of tumor type and location**

**Table d: Sub-analysis excluding patients less than 2 years old**

**Table e: Missing data impact on model estimates**

**Table f: Proportional odds assumption**

**Table g: Proportional odds logistic regression with and without outlier**

**Table a: Overview of Glucocorticoid types and conversion factor applied in this study (**excluding outliers and multiple types of Glucocorticoids administered**)**

| **Type of Glycocorticoid (**conversion factor per 5 Prednisolone equivalents**)** | **Preoperative Glucocorticoids (**n=499**)** | **Intraoperative Glucocorticoids (**n=291**)** |
| --- | --- | --- |
| **Betamethasone (**0.75**)** | 137 (27%) | 23 (8%) |
| **Dexamethasone (**0.75**)** | 342 (69%) | 251 (86%) |
| **Prednisolone (**5: reference**)** | 5 (1%) | 1 (0.3%) |
| **Methylprednisolone (**4**)** | 15 (3%) | 14 (5%) |
| **Hydrocortisone (**20**)** | 0 | 2 (0.7%) |

**Table b: Proportional odds logistic regression analysis for pGC, iGC and POSI with estimates of confounding variables**

|  | **Uniaspect analysis**  **n= 605 (pGC)**  **n= 564 (iGC)** | | **Model 1^a^**  **n=549** | | **Model 2^b^**  **n=485** | | **Model 3^c^**  **n=485** | |
| --- | --- | --- | --- | --- | --- | --- | --- | --- |
|  | **OR**  **(95% CI)** | **p-value** | **OR**  **(95% CI)** | **p-value** | **OR**  **(95% CI)** | **p-value** | **OR**  **(95% CI)** | **p-value** |
| **pGC** |  |  |  |  |  |  |  |  |
| No pGC given | 1.16  (0.56–2.43) | 0.69 | 1.06  (0.47–2.38) | 0.89 | 1.71  (0.69–4.27) | 0.24 | 1.06  (0.46–2.49) | 0.89 |
| Pr. doubling in dose prednisolone equivalent maximum dose pr. Day (mg/kg/day) | 1.69  (1.17–2.46) | 0.01 | 1.59  (1.06–2.42) | 0.03 | 1.80  (1.12–2.91) | 0.01 | 1.28  (0.84–1.98) | 0.25 |
| **iGC** |  |  |  |  |  |  |  |  |
| No iGC given | 1.61  (0.79–3.32) | 0.19 | 1.46  (0.67–3.19) | 0.34 | 1.11  (0.47–2.65) | 0.82 | 1.28  (0.58–2.82) | 0.53 |
| Pr. doubling in prednisolone equivalent dose  (mg/kg) | 1.49  (0.89–2.48) | 0.13 | 1.34  (0.76–2.35) | 0.31 | 0.93  (0.49–1.75) | 0.03 | 1.07  (0.62–1.82) | 0.79 |

| **Tumour type** |  |  |  |  |  |  |  |  |
| --- | --- | --- | --- | --- | --- | --- | --- | --- |
| Pilocytic/pilomyxoid  Astrocytoma |  |  |  |  | Ref = 1.00 |  | Ref = 1.00 |  |
| Medulloblastoma |  |  |  |  | 1.92  (1.15–3.25) |  | 1.85  (1.10–3.12) |  |
| Ependymoma |  |  |  |  | 1.05  (0.49–2.22) |  | 0.89  (0.41–1.88) |  |
| AT/RT |  |  |  |  | 2.99  (0.95–9.02) |  | 2.11  (0.64–6.63) |  |
| Other |  |  |  |  | 1.56  (0.70–3.31) |  | 1.85  (0.83–3.97) |  |
|  |  |  |  |  |  | 0.05 |  | 0.06 |
| **Tumour location** |  |  |  |  |  |  |  |  |
| Brainstem |  |  |  |  | Ref = 1.00 |  | Ref = 1.00 |  |
| Fourth Ventricle |  |  |  |  | 1.27  (0.75–2.16) |  | 1.15  (0.68–1.94) |  |
| Cerebellar Vermis |  |  |  |  | 0.31  (0.16–0.58) |  | 0.32  (0.16–0.59) |  |
| Cerebellar Hemisphere |  |  |  |  | 0.15  (0.07–0.32) |  | 0.16  (0.07–0.32) |  |
|  |  |  |  |  |  | <0.001 |  | <0.001 |
| **Age** |  |  |  |  |  |  |  |  |
| Pr. 1 year increase in age |  |  |  |  |  |  | 0.93  (0.88–0.99) | 0.02 |

Data are in OR (95% CI) and p-values. All dose values were log_2_ transformed. ^a^Model 1 is adjusted for pGC and iGC. ^b^Model 2 is adjusted for pGC, iGC, tumour type and tumour location. ^c^Model 3 is adjusted for pGC, iGC, tumour type, tumour location and age.

**Table c: Step-wise inclusion of tumor type and location**

|  | **Model 1^a^**  **n=549** | | **Model 2.1^b^**  **n=510** | | **Model 2.2^c^**  **n=** | | **Model 2^d^**  **n=** | |
| --- | --- | --- | --- | --- | --- | --- | --- | --- |
|  | **OR**  **(95% CI)** | **p-value** | **OR**  **(95% CI)** | **p-value** | **OR**  **(95% CI)** | **p-value** | **OR**  **(95% CI)** | **p-value** |
| **pGC** |  | |  | |  | |  | |
| No pGC given | 1.06  (0.47–2.38) | 0.89 | 1.03  (0.44–2.41) | 0.95 | 1.53  (0.64–3.68) |  | 1.71  (0.69–4.27) | 0.24 |
| Pr. doubling in dose prednisolone equivalent maximum dose pr. Day (mg/kg/day) | 1.59  (1.06–2.42) | 0.03 | 1.44  (0.93–2.24) | 0.11 | 1.68  (1.06–2.66) |  | 1.80  (1.12–2.91) | 0.01 |
| **iGC** |  | |  | |  | |  | |
| No iGC given | 1.46  (0.67–3.19) | 0.34 | 1.25  (0.55–2.87) | 0.59 | 1.11  (0.49–2.53) |  | 1.11  (0.47–2.65) | 0.82 |
| Pr. doubling in  prednisolone equivalent dose  (mg/kg) | 1.34  (0.76–2.35) | 0.31 | 1.12  (0.61–2.03) | 0.72 | 0.98  (0.54–1.78) |  | 0.93  (0.49–1.75) | 0.03 |

| **Tumour type** |  |  |  |  |  |  |  | |
| --- | --- | --- | --- | --- | --- | --- | --- | --- |
| Pilocytic/pilomyxoid  Astrocytoma |  |  | Ref = 1.00 | |  |  | Ref = 1.00 | |
| Medulloblastoma |  |  | 3.23  (2.04–5.18) |  |  |  | 1.92  (1.15–3.25) |  |
| Ependymoma |  |  | 2.59  (1.27–5.15) |  |  |  | 1.05  (0.49–2.22) |  |
| AT/RT |  |  | 5.65  (1.96–15.92) |  |  |  | 2.99  (0.95–9.02) |  |
| Other |  |  | 1.80  (0.86–3.63) |  |  |  | 1.56  (0.70–3.31) |  |
|  |  |  |  | <0.001 |  |  |  | 0.05 |
| **Tumour location** |  |  |  | |  | |  | |
| Brainstem |  |  |  |  | Ref = 1.00 | | Ref = 1.00 | |
| Fourth Ventricle |  |  |  |  | 1.07  (0.66–1.74) |  | 1.27  (0.75–2.16) |  |
| Cerebellar Vermis |  |  |  |  | 0.25  (0.13–0.44) |  | 0.31  (0.16–0.58) |  |
| Cerebellar Hemisphere |  |  |  |  | 0.12  (0.06–0.24) |  | 0.15  (0.07–0.32) |  |
|  |  |  |  |  |  |  |  | <0.001 |

Data are in OR (95% CI). All dose values were log_2_ transformed.

^a^Model 1 is adjusted for pGC and iGC.

^b^Model 2.1 is adjusted for pGC, iGC and tumour type

^c^Model 2.2 is adjusted for pGC, iGC and tumour location.

^d^Model 2 is adjusted for pGC, iGC, tumour type and tumour location**.**

**Table d: Sub analysis excluding patients less than 2 years old**

|  | **Model 3^a^**  **n=485** | | **Model 3**  **Without patients younger than**  **2 years**  **n=442** | |
| --- | --- | --- | --- | --- |
|  | **OR**  **(95% CI)** | **p-value** | **OR**  **(95% CI)** | **p-value** |
| **pGC** |  | |  | |
| No pGC given | 1.50  (0.60–3.79) | 0.39 | 1.34  (0.50–3.63) | 0.56 |
| Pr. doubling in dose prednisolone equivalent maximum dose pr. Day (mg/kg/day) | 1.59  (0.97–2.60) | 0.07 | 1.43  (0.84–2.45) | 0.19 |
| **iGC** |  | |  | |
| No iGC given | 0.98  (0.40–2.38) | 0.96 | 0.78  (0.30–2.01) | 0.61 |
| Pr. doubling in prednisolone equivalent dose  (mg/kg) | 0.84  (0.43–1.60) | 0.60 | 0.75  (0.35–1.51) | 0.42 |

Data are in OR (95% CI). All dose values were log_2_ transformed. ^a^Model 3 pGC, iGC, tumour type, tumour location and age.

**Table e: Missing data impact on pGC and IGC model estimates**

|  | **Uniaspect analysis** | | | | **Model 1^a^** | | | | | | | | **Model 2^b^** | |
| --- | --- | --- | --- | --- | --- | --- | --- | --- | --- | --- | --- | --- | --- | --- |
|  | **n= 605(pGC)**  **n= 564 (iGC)** | | **With data on both**  **pGC and**  **iGC**  **n= 549** | | **n=549** | | **With data on tumour type**  **n=510** | | **With data on tumour location**  **n=517** | | **With data on both**  **tumour type and location**  **n=485** | | **n=485** | |
|  | **OR**  **(95% CI)** | **p-value** | **OR**  **(95% CI)** | **p-value** | **OR**  **(95% CI)** | **p-value** | **OR**  **(95% CI)** | **p-value** | **OR**  **(95% CI)** | **p-value** | **OR**  **(95% CI)** | **p**  **value** | **OR**  **(95% CI)** | **p-value** |
| **pGC** |  |  |  |  |  |  |  |  |  |  |  |  |  |  |
| No pGC given | 1.16  (0.56–2.43) | 0.69 | 1.19  (0.55–2.59) | 0.65 | 1.06  (0.47–2.38) | 0.89 | 1.14  (0.50–2.62) | 0.76 | 1.11  (0.49–2.53) | 0.81 | 1.21  (0.52–2.83) | 0.66 | 1.71  (0.69–4.27) | 0.24 |
| Pr. doubling in dose prednisolone equivalent Max. Dose pr. Day (mg/kg/day) | 1.69  (1.17–2.46) | 0.01 | 1.71  (1.16–2.53) | 0.01 | 1.59  (1.06–2.42) | 0.03 | 1.62  (1.06–2.49) | 0.03 | 1.49  (0.97–2.29) | 0.07 | 1.56  (1.01–2.42) | 0.047 | 1.80  (1.12–2.91) | 0.01 |
| **iGC** |  |  |  |  |  |  |  |  |  |  |  |  |  |  |
| No iGC given | 1.61  (0.79–3.32) | 0.19 | 1.67  (0.81–3.53) | 0.17 | 1.46  (0.67–3.19) | 0.34 | 1.40  (0.63–3.13) | 0.41 | 1.52  (0.69–3.37) | 0.30 | 1.44  (0.64–3.27) | 0.37 | 1.11  (0.47–2.65) | 0.82 |
| Pr. doubling in prednisolone equivalent Max. Dose (mg/kg) | 1.49  (0.89–2.48) | 0.13 | 1.60  (0.94–2.70) | 0.08 | 1.34  (0.76–2.35) | 0.31 | 1.29  (0.72–2.30) | 0.39 | 1.33  (0.74–2.36) | 0.34 | 1.27  (0.70–2.29) | 0.43 | 0.93  (0.49–1.75) | 0.83 |

Data are in OR (95% CI) and p-values. All dose values were log_2_ transformed. Adjusting for age did not result in further exclusion of patients due to missing data and is omitted from this table.

^a^Model 1 is adjusted for pGC and iGC. ^b^Model 2 is adjusted for pGC, iGC, tumour type and tumour location.

**Table f: Proportional odds assumption**

|  |  | **Uniaspect analysis**  **n= 605 (pGC)**  **n= 564 (iGC)** | **Model 1^a^**  **n=549** | **Model 2^b^**  **n=485** | **Model 3^c^**  **n=485** |
| --- | --- | --- | --- | --- | --- |
|  |  | **OR**  **(95% CI)** | **OR**  **(95% CI)** | **OR**  **(95% CI)** | **OR**  **(95% CI)** |
| **pGC** |  |  |  |  |  |
| No pGC given | Proportional odds | 1.16  (0.56–2.43) | 1.06  (0.47–2.38) | 1.71  (0.69–4.27) | 1.50  (0.60–3.79) |
|  | 0 vs. 1 or 2 | 1.22  (0.58–2.58) | 1.09  (0.48–2.47) | 1.91  (0.75–4.93) | 1.68  (0.65–4.41) |
|  | 0 or 1 vs. 2 | 0.98  (0.36–2.64) | 0.93  (0.31–2.81) | 1.36  (0.39–4.75) | 1.17  (0.33–4.12) |
| Pr. doubling in prednisolone equivalent Dose pr. Day (mg/kg/day) | Proportional odds | 1.69  (1.17–2.46) | 1.59  (1.06–2.42) | 1.80  (1.12–2.91) | 1.59  (0.97–2.60) |
|  | 0 vs. 1 or 2 | 1.76  (1.20–2.59) | 1.63  (1.07–2.51) | 1.94  (1.18–3.23) | 1.73  (1.03–2.91) |
|  | 0 or 1 vs. 2 | 1.48  (0.90–2.40) | 1.41  (0.81–2.44) | 1.52  (0.79–2.90) | 1.28  (0.66–2.49) |
| **iGC** |  |  |  |  |  |
| No iGC given | Proportional odds | 1.61  (0.79–3.32) | 1.46  (0.67–3.19) | 1.11  (0.47–2.65) | 0.98  (0.40–2.38) |
|  | 0 vs. 1 or 2 | 1.65  (0.79–3.51) | 1.53  (0.68–3.51) | 1.17  (0.47–2.96) | 1.04  (0.41–2.68) |
|  | 0 or 1 vs. 2 | 1.64  (0.63–4.42) | 1.45  (0.52–4.21) | 0.96  (0.29–3.14) | 0.83  (0.24–2.80) |
| Pr. doubling in prednisolone equivalent (mg/kg) | Proportional odds | 1.49  (0.89–2.48) | 1.34  (0.76–2.35) | 0.93  (0.49–1.75) | 0.84  (0.43–1.60) |
|  | 0 vs. 1 or 2 | 1.49  (0.87–2.56) | 1.37  (0.75–2.50) | 0.98  (0.49–1.94) | 0.89  (0.44–1.78) |
|  | 0 or 1 vs. 2 | 1.68  (0.84–3.21) | 1.51  (0.71–3.12) | 0.89  (0.37–2.02) | 0.78  (0.31–1.83) |

| **Tumour type** |  |  |  |  |  |
| --- | --- | --- | --- | --- | --- |
| Medulloblastoma | Proportional odds |  |  | 1.92  (1.15–3.25) | 1.85  (1.10–3.12) |
|  | 0 vs. 1 or 2 |  |  | 1.92  (1.13–3.28) | 1.85  (1.09–3.18) |
|  | 0 or 1 vs. 2 |  |  | 1.75  (0.84–3.75) | 1.66  (0.80–3.59) |
| Ependymoma | Proportional odds |  |  | 1.05  (0.49–2.22) | 0.88  (0.40–1.89) |
|  | 0 vs. 1 or 2 |  |  | 0.99  (0.45–2.09) | 0.82  (0.37–1.79) |
|  | 0 or 1 vs. 2 |  |  | 1.32  (0.49–3.42) | 1.02  (0.37–2.72) |
| AT/RT | Proportional odds |  |  | 2.99  (0.95–9.02) | 2.14  (0.65–6.76) |
|  | 0 vs. 1 or 2 |  |  | 3.42  (1.02–11.84) | 2.48  (0.70–8.97) |
|  | 0 or 1 vs. 2 |  |  | 2.41  (0.47–9.94) | 1.48  (0.27–6.58) |
| Other | Proportional odds |  |  | 1.56  (0.70–3.31) | 1.79  (0.80–3.85) |
|  | 0 vs. 1 or 2 |  |  | 1.70  (0.75–3.74) | 1.93  (0.84–4.31) |
|  | 0 or 1 vs. 2 |  |  | 0.99  (0.26–3.15) | 1.20  (0.31–3.90) |

| **Tumour location** |  |  |  |  |  |
| --- | --- | --- | --- | --- | --- |
| Fourth Ventricle | Proportional odds |  |  | 1.27  (0.75–2.16) | 1.27  (0.74–2.16) |
|  | 0 vs. 1 or 2 |  |  | 1.33  (0.76–2.31) | 1.32  (0.76–2.31) |
|  | 0 or 1 vs. 2 |  |  | 1.18  (0.60–2.27) | 1.17  (0.60–2.26) |
| Cerebellar Vermis | Proportional odds |  |  | 0.31  (0.16–0.58) | 0.31  (0.16–0.57) |
|  | 0 vs. 1 or 2 |  |  | 0.34  (0.18–0.65) | 0.34  (0.17–0.64) |
|  | 0 or 1 vs. 2 |  |  | 0.09  (0.01–0.31) | 0.09  (0.01–0.31) |
| Cerebellar Hemisphere | Proportional odds |  |  | 0.15  (0.07–0.32) | 0.16  (0.07–0.33) |
|  | 0 vs. 1 or 2 |  |  | 0.16  (0.07–0.34) | 0.17  (0.07–0.34) |
|  | 0 or 1 vs. 2 |  |  | 0.08  (0.01–0.29) | 0.08  (0.01–0.30) |
| **Age pr. year** |  |  |  |  |  |
|  | Proportional odds |  |  |  | 0.94  (0.88–0.99) |
|  | 0 vs. 1 or 2 |  |  |  | 0.94  (0.89–1.00) |
|  | 0 or 1 vs. 2 |  |  |  | 0.92  (0.84–1.00) |

Data are in OR (95% CI). Outcome levels of speech were habitual speech (0), reduced speech (1) or mutism (2). Data are in OR(95% CI).

^a^Model 1 is adjusted for pGC and iGC. ^b^Model 2 is pGC, iGC, tumour type and tumour location. ^c^Model 3 pGC, iGC, tumour type, tumour location and age.

**Table g: Analysis with and without outlier**

|  | **Model 3 ^c^**  **With outliers**  **n=492** | | **Model 3**  **Without outliers**  **n=485** | |
| --- | --- | --- | --- | --- |
|  | **OR**  **(95% CI)** | **p-value** | **OR**  **(95% CI)** | **p-value** |
| **pGC** |  |  |  |  |
| No pGC given | 1.06  (0.46–2.49) | 0.89 | 1.50  (0.60–3.79) | 0.39 |
| Pr. doubling in dose prednisolone equivalent maximum dose pr. Day (mg/kg/day) | 1.28  (0.84–1.98) | 0.25 | 1.59  (0.97–2.60) | 0.07 |
| **iGC** |  |  |  |  |
| No iGC given | 1.28  (0.58–2.82) | 0.53 | 0.98  (0.40–2.38) | 0.96 |
| Pr. doubling in prednisolone equivalent dose  (mg/kg) | 1.07  (0.62–1.82) | 0.79 | 0.84  (0.43–1.60) | 0.60 |

| **Tumour type** |  | |  | |
| --- | --- | --- | --- | --- |
| Pilocytic/  pilomyxoid  Astrocytoma | Ref = 1.00 | | Ref= 1.00 | |
| Medulloblastoma | 1.85  (1.10–3.12) |  | 1.85  (1.10–3.12) |  |
| Ependymoma | 0.89  (0.41–1.88) |  | 0.88  (0.40–1.89) |  |
| AT/RT | 2.11  (0.64–6.63) |  | 2.14  (0.65–6.76) |  |
| Other | 1.85  (0.83–3.97) |  | 1.79  (0.80–3.85) |  |
|  |  | 0.06 |  | 0.06 |
| **Tumour location** |  | |  | |
| Brainstem | Ref = 1.00 | |  | |
| Fourth Ventricle | 1.15  (0.68–1.94) |  | 1.27  (0.74–2.16) |  |
| Cerebellar Vermis | 0.32  (0.16–0.59) |  | 0.31  (0.16–0.57) |  |
| Cerebellar Hemisphere | 0.16  (0.07–0.32) |  | 0.16  (0.07–0.33) |  |
|  |  | <0.001 |  | <0.001 |
| **Age** |  | |  | |
| Pr. 1 year increase in age | 0.93  (0.88–0.99) | 0.02 | 0.94  (0.88–0.99) | 0.03 |

Data are in OR (95% CI) and p-values. All dose values were log_2_ transformed.

^c^Model 3 includes pGC, iGC, tumour type, tumour location and age.

Outliers were defined as values greater than or less than 3 times the standard deviation from the mean of the log2 transformed doses.

The non-transformed doses of the outliers were preoperative doses (0.01, 0.11, 0.13, 0.18, 0.19, 55.71 mg/kg) and for intraoperative doses (0.01, 0.10, 11.11, 19.23, 22.22 mg/kg)
